# Fungal – assisted microalgae flocculation and simultaneous lignocellulolytic enzyme production in wastewater treatment systems

**DOI:** 10.1016/j.btre.2025.e00875

**Published:** 2025-01-10

**Authors:** Anna Civzele, Linda Mezule

**Affiliations:** Water Systems and Biotechnology Institute, Riga Technical University, Latvia

**Keywords:** Bioflocculation, Cellulolytic enzymes, Microalgae harvesting, Wastewater, White rot fungi

## Abstract

The study investigates the application of white rot fungi for reactor–scale microalgae harvesting and explores the mechanisms underlying the algal–fungal interactions and their impact on biomass composition. Enzymatic analysis and microscopy revealed that the formation of algal-fungal complexes and successful harvesting are coupled with fungal cellulose-degrading enzyme production and hydrolytic processes of microalgae cells. Fluorescence intensity decreased by over 80 % in cells stained with Calcofluor-white after interaction with white rot fungi, indicating the reduction in cellulose content in microalgal cells caused by fungal enzymatic activity. These enzymes also caused significant cell damage and more than 50 % decrease in microalgae cell size. The presence of cellulolytic enzymes broadens the potential application of the resulting biomass in various biotechnological applications. Moreover, reactor-scale bioflocculation resulted in over 95 % *T. obliquus* and almost 85 % *C. vulgaris* harvesting efficiency from secondary wastewater within less than 24 h, demonstrating the method's scalability and industrial applicability.

## Highlights

• Fungal-driven flocculation is a chemical-free, scalable algae harvesting method.
• Algal-fungal complexes form, aided by fungal enzyme production and hydrolysis.
• Algal-fungal interaction is more of a harmful relationship than symbiosis.
• Up to 95 % of microalgae were harvested from wastewater in 24 h at reactor scale.
• Resulting biomass can be used for cellulolytic enzyme production.

## Graphical abstract

**Figure:**
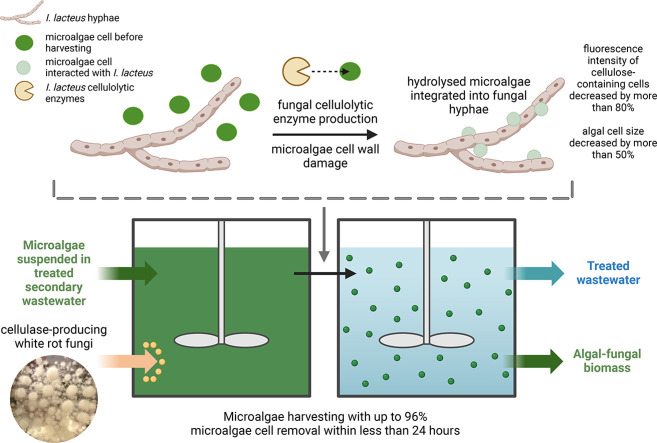

## 1 Introduction

For many years, microalgae have been regarded as a promising source of renewable biomass with a high content of valuable compounds, including proteins, carbohydrates, lipids, and bioactive compounds, making them a valuable resource for various industries, including biofuels, food, feed, and pharmaceuticals [1,2]. Moreover, microalgae have demonstrated potential in enhancing wastewater quality via the ability to efficiently remove nutrients, such as nitrogen and phosphorus, along with other pollutants [[3], [4], [5]]. Microalgae-based wastewater treatment technologies are also characterized by lower energy requirements compared to conventional wastewater treatment [6] and results in the production of algal biomass that can biologically reduce greenhouse gas emissions given that microalgae are able to capture carbon dioxide [7]. This biomass can also be utilized for biofuel production or synthesis of high-value bioactive compounds, contributing to a more circular and resource-efficient economy [8]. In contrast, huge amount of sludge is generated during the conventional wastewater treatment process, while sludge treatment and disposal are costly and can make up to 60 % of overall process costs [9].

However, the microalgae biomass harvesting still remains a critical bottleneck in the commercialization of microalgae technologies in industrial processes, including wastewater treatment, due to the microalgae cell size (less than 30 μm in diameter), low concentration distribution of cells, intercellular electrostatic repulsion and elevated growth rate [10]. Currently, microalgae harvesting methods primary include centrifugation, filtration, and chemical coagulation/flocculation [11]. However, despite the high efficiency, these technologies are mostly energy-intensive and costly [11,12], and may result in the contamination of harvested biomass and treated wastewater [13,14]. Moreover, centrifugation and filtration methods can potentially lead to cell damage and require substantial freshwater consumption, limiting their sustainability [15].

Biological harvesting techniques hold the potential to be the eco-friendly alternative for the physical and chemical-based harvesting methods [14]. The biological technologies rely on the microalgae aggregation process induced by the presence of microorganisms or their biopolymers [16,17]. Filamentous fungi have been studied as promising microalgae biological flocculants, showcasing not only efficient microalgae removal [18,19], but also the potential for simultaneous wastewater treatment [20]. In our previous study, we reported that microalgae flocculation with filamentous fungi results in the formation of relatively large and durable algal-fungal pellets, which can range from 5 to 15 mm in diameter [21]. These pellets can be easily removed using simple harvesting techniques, such as the chemical – free sedimentation of sieving, reducing the energy requirements and enhancing the overall efficiency of the microalgae harvesting process. However, despite its well-demonstrated efficiency and reduced dependence on the use of chemicals and energy, fungal-assisted harvesting still faces challenges due to a lack of large-scale testing [16], limitations in biomass reuse post-wastewater treatment, and gaps in understanding the underlying mechanisms of the process [22]. Furthermore, filamentous fungi used for microalgae harvesting in several previous studies are pathogenic and produce toxic compounds synthesized by *Aspergillus flavus* and some common fungi species [23]. In this scenario, similarly to chemical flocculants, the use of these fungi for microalgae removal may lead to potential contamination of biomass and the harvesting medium with fungal spores and pose risks of its leakage into the environment.

In response to these challenges, the reactor-scale microalgae harvesting using *I. lacteus* as a biological flocculant was investigated to more efficiently mimic full-scale conditions. *I. lacteus*, selected for microalgae harvesting, is a white rot basidiomycete that typically grows on decaying wood and lignocellulosic materials, playing an important role in decomposition processes in forest ecosystems. As a white rot fungus, it is capable to produce diverse spectrum of enzymes, such as cellulases, laccases, peroxidases, to degrade complex lignocellulosic structures [24]. In contrast to chemical flocculants and pathogenic fungi, the use of *I. lacteus* for microalgae bio-flocculation provides environmental and human health benefits by minimizing the risk of wastewater secondary contamination after harvesting process [25], while also enhancing opportunities for biomass reuse. Given the enzymatic abilities of *I. lacteus*, the potential for biotechnological production of lignocellulose-degrading enzymes from the recycled microalgae biomass was explored. Furthermore, the impact of these enzymes and their role in the formation of algal-fungal complexes were investigated by fluorescence microscopy and scanning electron microscopy (SEM) examinations, and fungal enzyme activity analysis, providing a novel interpretation of processes and mechanisms accompanying the fungal-assisted flocculation.

## 2 Materials and methods

### 2.1 Microorganisms and culture conditions

In this study, *Tetradesmus obliquus* (CCAP 276/10) and *Chlorella vulgaris* (CCAP 211/11B) were selected as representative microalgae strains and well-documented candidates for evaluating harvesting efficiency in wastewater systems, considering their proven effectiveness in nutrient removal in wastewater treatment [26,27]. Prior microalgae harvesting experiments, the microalgae biomass was cultivated in 1000 mL Pyrex® bottles in BG-11 growth medium for 10 days at 25–27 °C. Continuous 10 L h^-1^ aeration and the blue-red spectrum fluorescent light (180 μmol m^2^ s^−1^ at a 16:8 h lighting regime) was provided during the cultivation.

White-rot fungi *Irpex lacteus* (Fr.) was cultured and maintained on Potato Dextrose Agar (Oxoid Ltd., Basingstoke, Hants, UK) at 4 – 5 °C. Prior biological flocculation tests, *I. lacteus* was prepared in 250 mL Erlenmeyer flasks in a culture medium containing 0.8 g KH_2_PO_4_, 0.4 g K_2_HPO_4_, 0.5 g MgSO_4_·7H_2_O, 2 g NH_4_NO_3_, 2 g yeast extract and 10 g glucose per L. The pH of the medium was adjusted to 5.3–5.5. *I. lacteus* was cultivated for 3 days in an orbital shaker (New Brunswick™ Innova® 43, Eppendorf Austria GmbH, Wien, Austria) at 150 rpm and 30 °C until fungal pellets have been formed.

### 2.2 Wastewater source

The secondary wastewater was collected at a biological wastewater treatment plant “Daugavgriva” (Riga, Latvia, PE > 100 000) after secondary settlers. The wastewater was filtered through a 0.45 µm cellulose-acetate filter to remove indigenous bacteria and microparticles. The concentrations of all quality parameters for the wastewater used in this study (*n* = 3) (Table S1) were provided by internal WWTP monitoring.

### 2.3 Experimental setup

#### 2.3.1 Batch-scale microalgae flocculation

To investigate the mechanisms of *I. lacteus* – assisted algae flocculation, batch-scale tests were performed. These tests involved comparing enzymatic activity and changes in reducing sugar content across the following samples: (1) pure algal suspension without fungal addition, (2) pure fungal culture without algae biomass, (3) algal suspension with added active fungal pellets, and (4) algal suspension with added inactivated fungal pellets.

In samples where fungi were used, the harvesting experiments were initiated by inoculating pre-cultured *I. lacteus* fungal pellets into the suspension at a fungi:algae mass ratio of 1:2. For the samples with inactivated fungal pellets, pre – cultured *I. lacteus* pellets were deactivated by autoclaving for 20 min at 121 °C.

All samples were prepared in Schott Duran 250 mL laboratory bottles, which were then placed on an orbital shaker (PSU-20i, Biosan, Riga, Latvia) at 150 rpm 20 °C. Samples of the suspension were collected for the determination of microalgae harvesting efficiency, reducing sugar concentration, and fungal enzyme activity. Experiments were performed in 3 independent repeats.

#### 2.3.2 Reactor-scale microalgae flocculation

Reactor – scale harvesting experiments were performed in a 6.2 L reactor (EDF – 5.5, Bioreactors.net, Riga, Latvia) equipped with a Rushton turbine type mixer with 6 blades, which ensured the stirring of the suspension, and with pH and temperature control using temperature detection probe (TP-206A-CF-H1141-L300, Turck, Mülheim, Germany) and pH sensor (EasyFerm Plus PHI K8 325, Hamilton, Reno, Nevada, United States). Bio-flocculation were initiated by adding pre-cultured fungal pellets of *I. lacteus* to the microalgae suspension with fungi:algae dry mass ratio of 1:2. The tests were conducted in BG-11 algal medium to assess microalgae harvesting under optimal conditions and in secondary wastewater to more accurately reflect real-world applications. Moreover, the optimal stirring intensity was determined by performing microalgae harvesting tests at 0, 25, 50, 100, and 150 rpm mixing rates.

All reactor-scale tests were performed at a temperature of 20–22 °C and pH 7.0 ± 0.2. The concentration of microalgae cells in the suspension was measured daily in three repetitions to determine the progress of microalgae harvesting. Each experiment performed within this study was assessed in 3 independent repeats.

### 2.4 Determination of flocculation efficiency

Determination of the reduction in microalgae concentration in the medium during bio-harvesting process was ensured by measuring microalgae cell concentration using a UV-visible Spectrophotometer (GENESYS 150, Thermo Fisher Scientific Inc., Waltham, MA, USA) at 680 nm absorbance wavelength. Microalgae cell concentration in the samples was calculated by measuring its absorption in a linear interval. The efficiency of the microalgae removal by biological flocculation was determined using Eq. (1):

(1) $$E\%\;=\;\frac{C_{0}\;-\;C}{C_{0}}\;\times \;100\%,$$

where

*E*%: is microalgae harvesting efficiency,
*C*_0_: (cell/mL) is initial microalgae cell concentration before harvesting,
*C*: (cell/mL) is microalgae cell concentration after harvesting.

For both microalgae strains individual calibration curves were developed to relate microalgae cell concentration in the suspension and relevant optical density.

Additionally, the obtained results of microalgae harvesting efficiency, determined using the spectrophotometric method, were validated by staining the collected microalgae suspension samples with 10 μg mL^-1^ DAPI (4′,6-diamidino- 2-phenylindole, Merck, Germany) for 5–10 min. Prior staining, the samples of known volume were filtered through a 25 mm-diameter 0.2 μm-pore–size filter (Polycarbonate Track- Etch Membrane, Sartorius, Germany). Microalgae cell concentrations were determined with epifluorescence microscopy (Ex: 340/380; Em:>425, dichromatic mirror 565 nm, Leica DM6000B, Germany) by counting of 20 random fields of view.

### 2.5 Analytical methods of bioflocculation mechanisms

#### 2.5.1 Determination of cellulolytic enzyme activity

The samples of microalgae harvesting media were analyzed following the cellulase activity colorimetric assay described in the Mangan et al. [28] study [28]. In brief, the cellulase activity was determined by the incubation of testing sample supernatant with a substrate solution containing β-glucosidase at 40 °C for 10 min. The reaction was terminated by adding the stopping reagent, 2 % w/v Tris solution (pH 9). The absorbance values were determined using a microplate reader (The CLARIOstar® Plus, BMG Labtech, Germany) at 400 nm against the time zero reading for the respective substrate. Data from the cellulase activity assays were analyzed to assess the significance of changes in enzyme activity over time.

In addition to assessing fungal cellulase activity, the concentration of reducing sugars in the microalgae suspension was determined using the dinitrosalicylic acid (DNS) method [29], which was adjusted for microplate scale for this study. In brief, 10 µL of the liquid sample was mixed with 10 µL of 0.05 M sodium citrate buffer and 60 µL of 3,5-dinitrosalicylic acid in the 96 well microplate. Distilled water was used as a control. All samples were heated at 100 °C for 5 min. Then, 220 mL of distilled water was added, and the absorption of the solution was measured using the microplate reader (CLARIOstar plus, BMG Labtech) at 540 nm. Prior reducing sugar determination, the standard curve of known sugar concentration was constructed to obtain absolute reducing sugar concentrations. The reducing sugar concentration was defined as the mg of reducing sugars per g of dry biomass (mg/g).

#### 2.5.2 Determination of ligninolytic enzyme activity

To detect the presence of fungal ligninolytic enzymes, laccase and manganese peroxidase activity analysis was performed. Laccase activity was determined by the ABTS method [30]. Oxidation of ABTS was measured by determining the increase in A436 (ε_436_ = 36,000 M^−1^ cm^−1^). The reaction mixture contained 10 mM ABTS, 0.1 M sodium acetate buffer (pH 4.5), and the supernatant of the collected sample. Absorbance was determined at 436 nm in a spectrophotometer (The CLARIOstar® Plus, BMG Labtech, Germany) against the distilled water as a blank.

Manganese peroxidase activity analysis was performed according to Kuwahara et al. [31]. The reaction mixture contained the collected sample solution, 50 mM sodium succinate buffer (pH 4.5), 50 mM sodium lactate (pH 5), 0.1 mM M MnSO_4_, 0.1 mM phenol red, and 100 μM H_2_O_2_. Absorbance was monitored at 610 nm using a spectrophotometer (The CLARIOstar® Plus, BMG Labtech, Germany) against the distilled water as a blank.

#### 2.5.3 Fluorescence microscopy

Microalgae cells, both before and after bioflocculation, were stained with Calcofluor-white (Sigma Aldrich, St. Louis, Missouri, US), which specifically binds to structures containing cellulose and chitin [32], to explore the possible effect of fungal cellulolytic enzymes produced during flocculation process. Sample visualization was made with epifluoresence microscopy (ZEISS Axioscope 5 equipped with fluorescence system, filter set 01 Ex BP 365/12 Em LP 397, Oberkochen, Germany). All images were acquired (Zeiss Axiocam 506, color) using an equal exposure time of 10 ms and analyzed with ZEN 3.2. software (ZEISS, Oberkochen, Germany).

#### 2.5.4 Scanning electron microscopy

To determine the potential changes in algae cell wall structure after fungal-assisted flocculation and the possible hydrolysis by fungal lignocellulolytic enzymes the microalgae cell morphology before and after harvesting was examined using the high-resolution SEM electron microscope Verius 5 UC (Thermo Scientific), operated at 1 kV, employing the Everhart-Thornley Detector and TLD (thru lens detector). Samples were left uncoated, and to prevent charging, a low current of 13 pA and a vector scanning approach with a dwell time of 50 ns were utilized. Prior observation, the samples were fixed with a 4 % formaldehyde for 24  h at 4  °C, then dehydrated in ethanol series (25 %, 50 %, 75 %, 95 % and 100 %, 10 min each), and subjected to critical-point drying (CPD, Leica EM CPD300, Germany) for 6 h

### 2.6 Lignocellulose-degrading enzyme production

To assess the potential of the fungal enzyme production, the harvested algal-fungal biomass was homogenized (1 min, 400 rpm, Retsch GM 200, Haan, Germany). Then the prepared biomass was used for inoculation in 250 mL Erlenmeyer flasks containing 0.8 g KH_2_PO_4_, 0.4 g K_2_HPO_4_, 0.5 g MgSO_4_·7H_2_O, 2 g NH_4_NO_3_, 2 g yeast extract and 4 % of hay biomass per L (pH 5.3–5.5) for 4 days in an orbital shaker (150 rpm, 30 °C, New Brunswick™ Innova® 43, Eppendorf Austria GmbH, Wien, Austria). Hay (dry weight (DW): 92.8 ± 1.3 %; ash 6.03 %, collected from semi-natural grassland in Latvia) was used as a carbon source for fungi to induce the release of lignocellulose-degrading enzymes. The adopted composition of the biomass was approximately 22–26 % cellulose, 14–25 % hemicellulose, and 1–13 % lignin [33].

After incubation, the liquid fraction was centrifuged for 10 min at 8500 RCF to remove the solids. Proteins were precipitated with 50 % w/v ammonium sulfate and stored at 4 °C for 24 h. The obtained enzyme sediments were extracted by centrifugation for 10 min at 8500 RCF. After centrifugation, the extracted enzyme was stored in 0.05M sodium citrate buffer at 4 °C.

Enzyme activity was analyzed according to a standard FPU method [29] and expressed as filter paper units (FPU) per mL of produced enzyme. The cellulase activity was also determined by previously described cellulase activity assay prepared by Mangan et al. [28].

### 2.7 Analysis of algal-fungal biomass composition

The composition of resulting biomass obtained after microalgae flocculation tests was analyzed to determine the concentration of the valuable compounds – carbohydrates, lipids, and proteins, compared to pure algal biomass of *T. obliquus* and *C. vulgaris*.

#### 2.7.1 Determination of total carbohydrate concentration

The phenol-sulfuric acid method [34] was used for total carbohydrate concentration measurements. To determine the total carbohydrate concentration, 0.1 g of dry biomass samples were hydrolyzed by adding 3 % sulfuric acid (H_2_SO_4_) and autoclaving at 121 °C for 20 min. The absorption of the resulting solution was measured using a UV spectrophotometer (GENESYS 150, Thermo Fisher Scientific Inc., Waltham, MA, USA) at an absorption wavelength of 490 nm.

#### 2.7.2 Determination of lipid content

Sulfo-phospho-vanillin method was used to determine the lipid content [35]. For sample preparation, 1 mL of the tested biomass solution with a concentration of 1–3 g/L was centrifuged at 2000 RCF for 10 min. Next, the supernatant was separated, and the precipitated biomass sample was dissolved in 1 ml of concentrated sulfuric acid (H_2_SO_4_). 100 μL of H_2_SO_4_ was added to 100 μL of the prepared sample. The sample was mixed thoroughly and incubated for 10 min at 100 °C. After the incubation time, 500 μL of phosphor-vanillin reagent was added, and the sample was incubated for 15 min at 37 °C. Lipid content was determined by measuring the absorption of the obtained sample at 530 nm using a microplate reader (The CLARIOstar® Plus, BMG Labtech, Germany).

#### 2.7.3 Determination of protein content

Protein content in the biomass obtained after bioflocculation tests was determined by the Lowry method [36]. For this procedure, samples containing approximately 0.1 g of dry tested biomass were treated with a probe sonicator for 2 min (130 W, 20 KHz, 30 % amplitude, Cole-Parmer, USA). After treatment, the samples were diluted to 20 mL with phosphate buffered saline solution (PBS) (pH 7.4) and incubated in a water bath at 100 °C for 15 min. After incubation, the samples were centrifuged at 10,000 RCF for 15 min. The obtained supernatant was used for the analysis using a UV-visible Spectrophotometer (GENESYS 150, Thermo Fisher Scientific Inc., Waltham, MA, USA) at 750 nm absorbance wavelength. Bovine serum albumin (BSA, Sigma Aldrich, St. Louis, Missouri, US) was used as the reference compound.

## 3 Results and discussion

### 3.1 Reactor – scale microalgae bioflocculation

Various harvesting techniques have demonstrated high efficiency at the batch-scale, yet there still remains a lack of well-defined methods for achieving equally efficient large – scale harvesting [16,37]. The main concerns associated with microalgae harvesting include the potentially high operational costs of the process when transitioning from batch to reactor or pilot scale systems, along with possible variations in the harvesting efficiency [14].

**Fig. 1:**
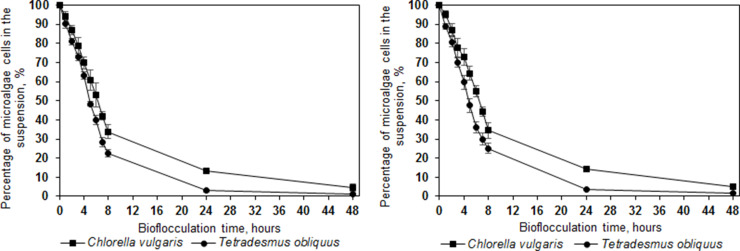
Percentage of microalgae cells remaining in suspension when bio-flocculated with *I. lacteus* in algal medium (left) and wastewater collected after secondary settlers (right). Standard deviation represents the average value from three independent repeats.

Within this study, during the reactor-scale tests, *T. obliquus* cell concentration in the artificial growth media was reduced by 97.09 ± 0.78 % and by 96.41 ± 0.49 % in the secondary wastewater. The concentration of *C. vulgaris* cells was reduced by 87.75 ± 0.96 % in the artificial growth media and by 84.68 ± 1.49 % in the secondary wastewater (Fig. 1). Moreover, the concentration of *T. obliquus* cells was reduced by 74.96 ± 2.65 % and by 65.54 ± 4.12 % for *C. vulgaris* cells within first 8 h. During this flocculation stage, a nearly linear trend of *T. obliquus* and *C. vulgaris* cell reduction (*R*^2^ was 0.9919 and 0.9917, respectively) was observed. If the reduction process of microalgae cells remains linear after 8 h, up to 95 % *T. obliquus* cell concentration reduction in secondary wastewater could possibly be achieved within 9.5 h, as well as more than 80 % of *C. vulgaris* cells could be removed within 10.7 h. The more time-efficient microalgae removal compared to previously stated 24 h can reduce the operational costs of microalgae harvesting in large scale processes, given the shorter time needed for maintaining optimal cultivation and harvesting conditions for the successful flocculation. Furthermore, reactor – scale microalgae harvesting did not demonstrate any significant difference in removal efficiency either when artificial growth medium or secondary wastewater was used (*p* > 0.05), showing the method's adaptability across different flocculation media and real conditions.

**Fig. 2:**
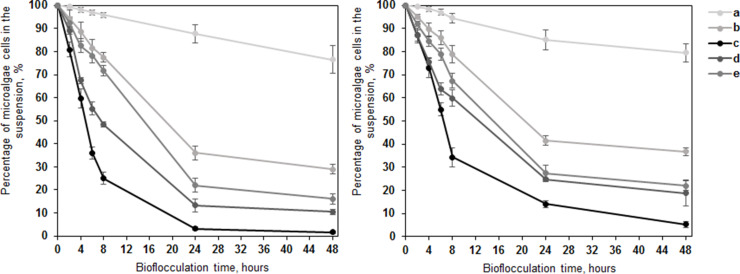
Percentage of *T. obliquus* (left) and *C. vulgaris* (right) cells remaining in suspension when bio-flocculated with *I. lacteus* at a) 0; b) 25; c) 50; d) 100 and e) 150 rpm mixing rate. Standard deviation represents the average value from three independent repeats.

In our previous research, batch-scale tests in artificial growth medium have demonstrated more than 95 % reduction in *T. obliquus* and 70 % reduction in *C. vulgaris* within 24 h of bioflocculation by *I. lacteus* [21]. In the secondary wastewater, 77 % to 82 % removal efficiency was obtained. In this study, the heightened efficiency observed in reactor-scale tests can be attributed to improved control over environmental conditions and enhanced stirring efficiency, promoting better dispersion of fungal pellets and microalgae biomass throughout the suspension. To determine the optimal stirring conditions for the studied flocculation process, stirring intensities, ranging from 0 to 150 rpm, and the impact on flocculation process were investigated (Fig. 2). The highest microalgae flocculation efficiency – more than 96 % removal of *T. obliquus* cells and more than 84 % reduction in *C. vulgaris* cells, was achieved at 50 rpm microalgae suspension mixing rate, determined as the most efficient mixing intensity mode.

The absence of microalgae suspension stirring (0 rpm) resulted in 81–85 % decrease in harvesting efficiency compared to the most successful flocculation results observed after 24 h. The highest efficiency under no-mixing conditions was significantly lower compared to the optimal stirring mode (*p* < 0.05). It was achieved after 96 h of flocculation and resulted in only a 40.88 ± 5.74 % reduction in *T. obliquus* cells and a 39.54 ± 5.13 % reduction in *C. vulgaris* cells. The major proportion of microalgae cells remained free floating due to the sedimentation of *I. lacteus* pellets at the bottom of the reactor in absence of proper mixing. A significant decrease in harvesting efficiency (*p* < 0.05) was also observed, when the flocculation process was performed at mixing intensity of 25 rpm.

A negative impact on the flocculation process was also observed when mixing rate was from 100 to 150 rpm. The flocculation efficiency of *T. obliquus* after 24 h was 86.79 ± 2.84 % at 100 rpm and 77.98 ± 3.05 % at 150 rpm. During the flocculation of *C. vulgaris*, 75.14 ± 0.66 % and 72.62 % ± 3.34 % of cells were removed after 24 h at 100 and 150 rpm respectively. Thus, the microalgae harvesting efficiency decreased on average from 10.30 % to 19.11 % due to excessive stirring compared to optimal conditions (50 rpm). Too vigorous agitation resulted in disintegration of added pre – cultured fungal pellets leading to poor accumulation of microalgae cells. Therefore, in large-scale systems, a balance must be achieved, where stirring is indispensable for optimal flocculation efficiency, yet excessive agitation needs to be avoided to ensure cost-effectiveness and process integrity.

Beyond stirring intensity, the choice of impeller type also may play a role in the microalgae flocculation and aggregate formation process. The Rushton turbine, used in this study, is the most widely used impeller in conventional bioreactors, inducing a radial flow in the broth [38]. However, impellers with axial or mixed (axial/radial) flows have been investigated to enhance circulation within the reactor, generating lower shear stress in microbial cultivation systems compared to radial flow impellers [39]. Therefore, the previously observed decrease in microalgae flocculation efficiency at higher stirring intensities may also be attributed to the damage of formed fungal pellets and microalgae flocs caused by the impeller blades. For a lower risk of algal – fungal floc damage, axial impellers, such as the Elephant Ear may be also suitable [38]. Thus, to achieve a more comprehensive understanding and improvement of the studied technology, the optimal impeller type, and its impact on the bio – flocculation process should be investigated.

### 3.2 Microalgae bioflocculation mechanisms

#### 3.2.1 Algal cell morphology observation

**Fig. 3:**
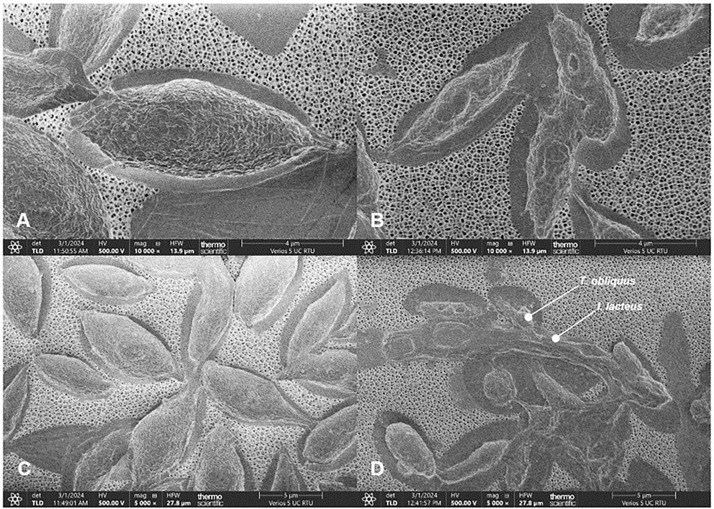
SEM images of *T. obliquus* microalgal cells: (a, c) before harvesting; (b) after 24 h of harvesting and interaction with *I. lacteus*; (d) attached to hyphae of *I. lacteus* after 24 h of harvesting.

To explore the mechanisms underlying the algal-fungal floc formation and the potential changes in algae cells caused by interaction with *I. lacteus*, the microalgal cells before and after fungal-assisted harvesting were explored using scanning electron microscopy (SEM). The obtained images demonstrated the changes in the size, structure, and integrity of cells between untreated microalgae and microalgae that underwent the fungal-assisted flocculation (Fig. 3). In contrast to the untreated algae, the observed algal cells that were harvested by *I. lacteus* exhibited signs of cell wall damage and had also undergone shrinkage in size. Furthermore, the microscopy examination revealed that the numerous microalgal cells were trapped or partly absorbed inside the fungal hyphae. This indicates on the presence of biological processes occurring during the algal-fungal interactions that lead to microalgae cell disruption. White rot fungi, known for degrading lignocellulosic compounds via enzymatic hydrolysis, may induce similar cell damage to algae during lignocellulosic biomass degradation. Therefore, the mechanisms of the studied process were further investigated by the determination of fungal enzyme activity as well as the examination of cellulose content in microalgae cells.

#### 3.2.2 Determination of fungal enzyme activity

**Fig. 4:**
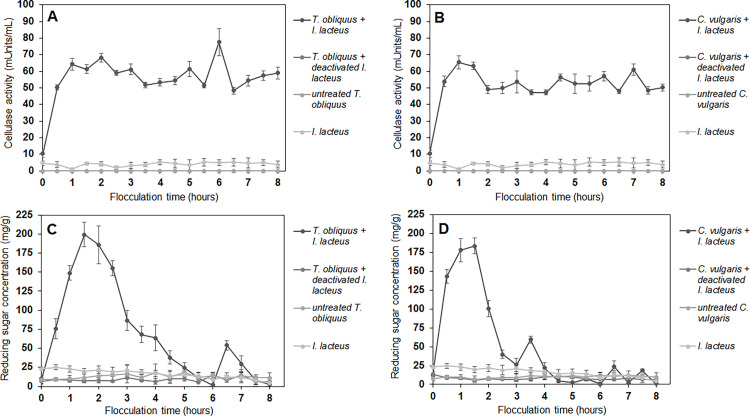
The dynamics of (a, b) fungal cellulase activity and (c, d) reducing sugar concentration during the interaction between *I. lacteus* and microalgae cells. Standard deviation represents the average value from three independent repeats.

To detect the potential cell-degrading processes occurring during the microalgae harvesting by fungi, the presence of fungal lignocellulolytic enzymes and their activity as well as the reducing sugar concentration were determined in the harvesting media during algal-fungal interaction. The rapid increase in the reducing sugar concentration in the first hours of co-cultivation was observed solely in the samples of the microalgae suspension with active fungal pellets (Fig. 4). The peak of reducing sugar concentration in the suspension of both microalgae strains was detected after 1.5 h after addition of *I. lacteus* pellets. The reducing sugar content was increased from the initial 10.01 ± 3.37 mg/g to 199 ± 16.08 mg/g in 1.5 h for *T. obliquus* – *I. lacteus* system, and from the initial 11.13 ± 3.30 mg/g to 183.73 ± 10.61 mg/g when *C. vulgaris* was flocculated. Conversely, no significant changes in reducing sugar content were observed in the samples of algal and fungal monocultures and in the algal suspension containing inactivated fungi (*p* > 0.05).

Interacting with the lignocellulosic biomass, *I. lacteus* releases ligninolytic enzymes to oxidize aromatic substrates, producing aromatic radicals and modifying the structure of the lignocellulose-containing raw materials and lignin to access the cellulose and hemicellulose [24]. Afterwards, by releasing cellulase enzymes, wood – decaying fungi break down cellulose, a complex carbohydrate also found in the cell walls of microalgae [40]. Given the enzymatic capabilities of *I. lacteus* as a white rot fungus and detected sugar dynamics in the first hours of the flocculation process, the presence of lignocellulose – degrading enzymes – laccase, manganese peroxidase (MnP), and cellulase was analyzed.

The enzymatic assay results revealed absence of laccase and MnP enzymatic activities in all samples, suggesting that these particular ligninolytic enzymes might not be actively involved in the microalgae flocculation process under the tested conditions. As microalgae cells are primarily composed of cellulose [40], fungal cellulase activity was considered a critical parameter. The production of cellulolytic enzymes was only detected when the harvesting process included active *I. lacteus* pellets, where the rise of the sugar concentration in the suspension immediately followed the fungal cellulase production. The first peak of the cellulase activity in both *T. obliquus* and *C. vulgaris* suspensions (64.24 ± 3.65 mUnits/mL and 65.41 ± 3.96 mUnits/mL, respectively) was detected 0.5 h before the maximal sugar production that was registered after 1 h of the flocculation (Fig. 4).

During the early stage of *T. obliquus* harvesting, the highest cellulase activity – 77.64 ± 8.18 mUnits/mL, was achieved after 6 h of fungal pellet introduction in the suspension. This increase of cellulase production resulted in repeated reducing sugar production – 53. 97 ± 6.16 mg/g after 6.5 h of flocculation process. Similarly, the release of reducing sugars was also observed after determined peaks of cellulase activity during *C. vulgaris* harvesting. Above 60 mUnits/mL cellulase activity was observed after 1 hour (65.41 ± 3.96 mUnits/mL), 1.5 h (63.20 ± 2.36 mUnits/mL) and 7 h (60.99 ± 3.31 mUnits/mL) of the *C. vulgaris* flocculation.

Furthermore, during these experiments, the successful removal of the microalgae cells from the suspension was also achieved only in the presence of active fungal pellets, where more than 75 % of *T. obliquus* and 65 % of *C. vulgaris* cells were harvested in 8 h and up to 97 % of *T. obliquus* and 89 % of *C. vulgaris* cells in 24 h. In contrast to the active fungi, in the co-culture with the inactivated fungi, only a 3.76 ± 1.23 % reduction in *T. obliquus* and a 2.40 ± 0.65 % reduction in *C. vulgaris* were achieved in 24 h, indicating the dependence of microalgae flocculation efficiency on the presence of fungal pellets with active enzyme-producing abilities.

The observed cellulase activity rise in the presence of active *I. lacteus* and subsequent sugar production suggest that the detected fungal enzyme group might cause the breakdown of cellulosic compounds during the interaction with microalgae biomass. These hydrolytic enzymes may cause the cell damage and shrinkage previously observed in SEM images, weaking the microalgae cell wall structure and contributing the cell accumulation process into the fungal mycelial network.

#### 3.2.3 Fluorescence microscopy examination

**Fig. 5:**
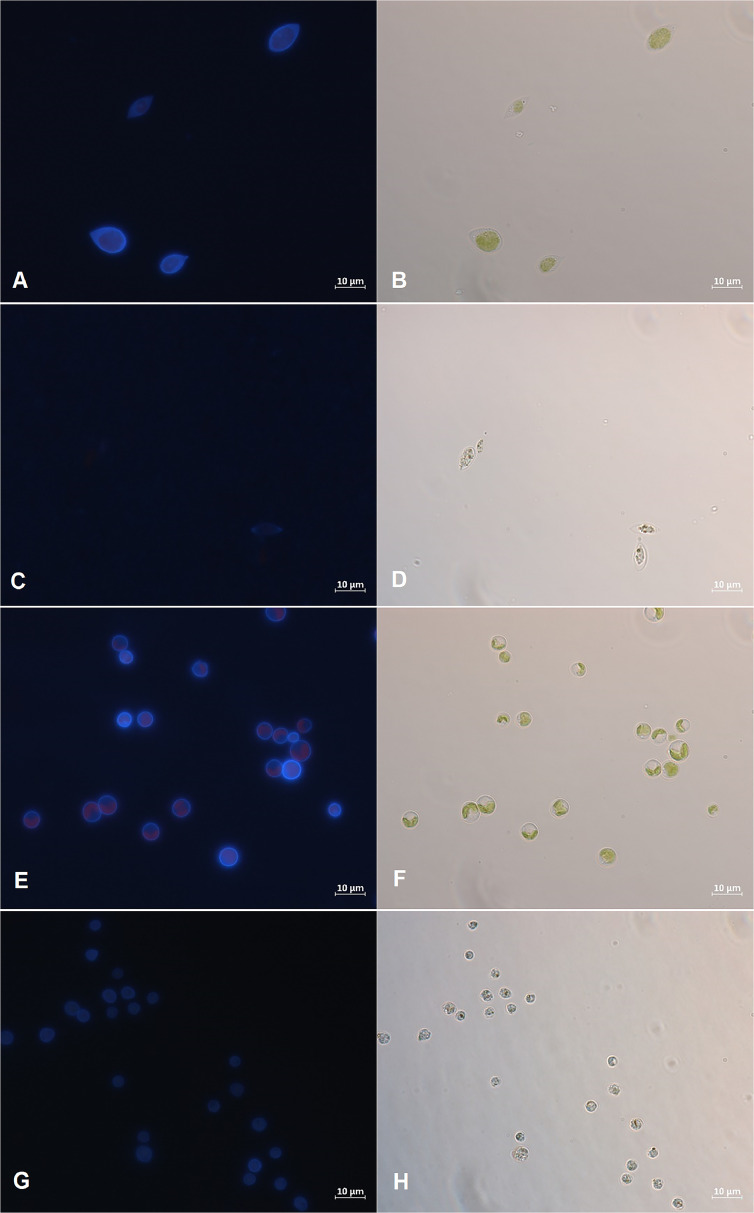
Microscopy images of (a, b, c, d) *T. obliquus* and (e, f, g, h) *Chlorella vulgaris* microalgal cells (a, b, e, f) before fungal-assisted harvesting; (c, d, g, h) after fungal-assisted harvesting.

To assess whether the observed reducing sugar production resulted from the cellulolytic enzyme activity and the breakdown of cellulose compounds, the cellulose content of microalgae cells was analyzed. After staining the cells with Calcofluor-white, the significant changes in fluorescence intensity (*p* < 0.05) between untreated microalgae and microalgae that underwent the fungal-assisted harvesting process were observed (Fig. 5) (see supplementary material). The fluorescence intensity of *T. obliquus* and *C. vulgaris* decreased by more than 85 % and 80 %, respectively, after 24 h of the interaction with active *I. lacteus*. As the Calcofluor-white binds to cellulose compounds, the observed changes in fluorescence intensity indicated the reduction in cellulose content in microalgal cells caused by the presence of the white rot fungus and the cellulolytic enzymes. Furthermore, the microscopy examination also revealed the significant changes in microalgal cell size after co-cultivation of microalgae and active fungal pellets (*p* < 0.05). After 24 h of harvesting, the area of *T. obliquus* cells decreased on average from 67.73 ± 17.74 µm to 26.54 ± 6.48 µm, and the area of *C. vulgaris* cells decreased from 30.96 ± 6.90 µm to 14.64 ± 5.30 µm. In contrast, the microalgae cell size and the fluorescence intensity of untreated microalgae and microalgae cells co-cultivated with inactivated fungal pellets did not change significantly (*p* > 0.05).

These results, combined with the SEM observations and the fungal enzyme activity analysis, propose a new understanding of the mechanisms behind fungi-induced microalgae harvesting. The role of cellulolytic enzymes in the algal cell accumulation process was confirmed by the absence of successful flocculation when enzymatically inactive fungi were used. Therefore, the results suggest that when active fungal mycelium is introduced into a microalgae suspension, the fungi interact with microalgal cells similarly to lignocellulose-containing biomass. Fungi may release cellulases that specifically target and disrupt the cell walls of microalgae by breaking down cellulose compounds, leading to the weakening of the algal cell structure and the release of cell wall polysaccharides and other cellular components. This hydrolytic process produces reducing sugars that fungi can utilize for their metabolism and facilitates the integration of the cells into fungal structures. Consequently, the cellulolytic enzyme production and the structural degradation of the algal cells promotes their aggregation into flocs, enhancing the efficiency of the harvesting process.

While previous studies suggested an efficient harvesting process is based on a symbiotic relationship between fungi and algae [16], this study revealed that the studied process is accompanied by the hydrolytic processes and damage to microalgae cells caused by fungal activity, suggesting that the observed microalgal-fungal interaction during harvesting can be classified more as a parasitic or harmful relationship rather than symbiosis. The obtained results also suggest that fungi with high cellulase production capabilities can appear to be more potent microalgae flocculants, given that highly efficient harvesting was possible only in the presence of cellulase-producing fungal pellets. These findings could facilitate the selection process of the most suitable fungal strain for the microalgae harvesting, addressing a current challenge associated with the strong dependence of efficiency of the studied technology on the used fungal strains [41]. The detection of fungal enzyme activity could also open the possible algal-fungal biomass reuse opportunities after wastewater treatment.

### 3.3 Recycling potential of fungal – algal biomass

#### 3.3.1 Harvested biomass composition analysis

One of the current limitations for microalgae flocculation with fungi is the potential impact on harvested biomass quality and undesirable changes in the biomass composition due to fungal integration [42]. The microalgae strain selection is not only based on the pollutant removal capabilities from the wastewater; it also considers the content of valuable compounds in the microalgal biomass [3,43]. Therefore, the changes in the harvested biomass composition can be critical to potential utilization in various fields, including biofuel production. While microalgae and filamentous fungi have been used to independently produce various bioproducts [8,44], the recycling opportunities for fungal – algal biomass remains to be explored.

**Fig. 6:**
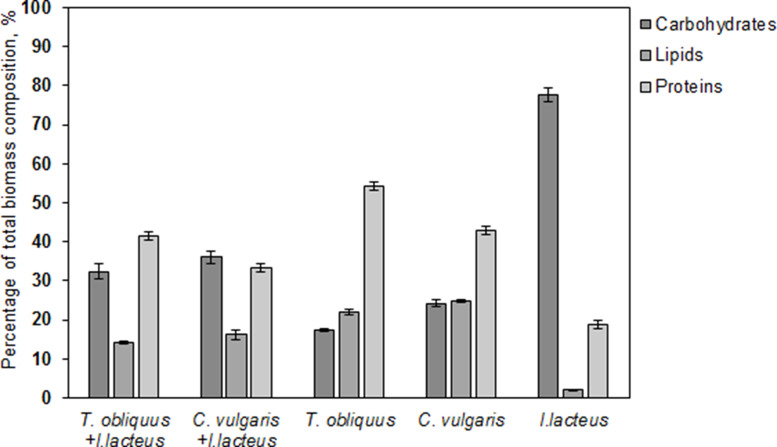
Comparative analysis of total carbohydrates, lipids and proteins in the algal-fungal biomass compared to pure algal and *I. lacteus* biomass composition. Standard deviation represents the average value from three independent repeats.

As the result of the co – cultivation of microalgae and fungal biomass, changes in the macromolecular composition were observed. The *T. obliquus – I. lacteus* complex exhibited a 14.91 % increase in carbohydrates compared to untreated *T. obliquus*. The *C. vulgaris - I. lacteus* complex had a 11.79 % increase in carbohydrates (Fig. 6). The introduction of *I. lacteus* to the protein – rich microalgae biomass consequently affected the protein concentration in fungal-algal biomass, which decreased by 12.68 % and 9.71 % compared to untreated *T. obliquus* and *C. vulgaris* biomass, respectively. This poses challenges, especially if the biomass is intended for applications requiring higher protein content. Additionally, lipid content in the biomass harvested by fungal-assisted flocculation was approximately 8 % lower compared to untreated algal biomass, which can influence the biomass potential in applications requiring lipid-rich biomass, such as biodiesel production [45]. Thereby, alternative biomass utilization opportunities were further explored, given the possible fluctuations in the content of valuable compounds.

#### 3.3.2 Lignocellulose-degrading enzyme production from algal-fungal biomass

Considering the observed fungal enzyme dynamics during microalgae harvesting, the potential of lignocellulose-degrading enzyme production was evaluated for the white rot fungal mycelium enriched with algal biomass. These enzymes produced by fungi can be used in the sustainable lignocellulose degradation to produce the energy, fuel, and valuable chemicals [46]. Thus, lignocellulolytic enzyme cocktail was produced using the harvested biomass after the successful microalgae flocculation, as well as by pure, individually cultivated *I. lacteus*.

The enzymatic analysis revealed a similar activity of the enzymes produced by both harvested algal-fungal biomass and pre-cultured fungal biomass. The activity of cellulase, produced by *I. lacteus, T. obliquus* – *I. lacteus* and *C. vulgaris* – *I. lacteus* complex, yielded values of 1061.66 ± 72.01 mUnits/mL, 952.04 ± 62.02 mUnits/mL, and 928.88 ± 69.77 mUnits/mL, respectively. When expressed in filter paper units (FPU) per mL of produced enzyme, the enzymatic activity was 8.31 ± 0.69 FPU per mL for pure *I. lacteus* biomass, while enzyme production using *T. obliquus* – *I. lacteus* and *C. vulgaris* – *I. lacteus* resulted in 7.71 ± 0.79 FPU per mL and 7.45 ± 0.61 FPU per mL, respectively.

Therefore, the studied method not only provides a sustainable and efficient approach to microalgae harvesting but also presents opportunities for recycling the harvested biomass in lignocellulose-degrading enzyme production. Given that fungal-assisted microalgae harvesting results in the generation of significant amount of algal-fungal biomass, this biomass can replace freshly cultivated fungi in enzyme production processes. This substitution could eliminate the need for a separate biomass production step in lignocellulolytic enzyme production, thereby reducing the costs associated with the prior cultivation of enzyme-producing fungi and the maintenance of optimal cultivation conditions.

Considering the activity of enzyme cocktail obtained from harvested biomass, the produced enzymes can be used to break down lignocellulosic biomass into fermentable sugars, which can further be converted into biofuels like ethanol [8]. Moreover, the fungi-enriched algal biomass and the produced lignocellulolytic enzymes can be utilized in composting and bioconversion processes to decompose plant waste materials [47], as well as in wastewater treatment, the paper and pulp industry, and the textile industry [48]. Therefore, even though the algal biomass composition can significantly change during fungal-assisted harvesting process, the presence of white rot fungi in the resulting biomass makes it a potential resource for a broad range of applications, addressing concerns regarding the use of fungi for microalgae harvesting.

## 4 Conclusions

Efficient microalgae harvesting was achieved solely in the presence of enzyme-producing *I. lacteus* pellets, resulting in 97 % of *T. obliquus* and 89 % of *C. vulgaris* removal from the wastewater within 24 h. The bioflocculation process was accompanied by the enzymatic hydrolysis of microalgae cells, indicating a potentially harmful relationship with the fungi rather than symbiosis. The interaction was supported by the reduction of algal cell size and cellulose content and the formation of reducing sugars, signifying the role of cellulolytic enzymes in fungal-assisted flocculation process. Moreover, the study demonstrates the method's scalability for wastewater treatment systems and the potential of harvested biomass in biotechnological applications.

## CRediT authorship contribution statement

**Anna Civzele:** Writing – review & editing, Writing – original draft, Visualization, Methodology, Investigation, Formal analysis, Conceptualization. **Linda Mezule:** Writing – review & editing, Writing – original draft, Supervision, Conceptualization.

## Declaration of competing interest

The authors declare that they have no known competing financial interests or personal relationships that could have appeared to influence the work reported in this paper.

## Data Availability

Data will be made available on request.

## Appendix Supplementary materials

Image, application 1
