# Supplementary material for: Fungal – assisted microalgae flocculation and simultaneous lignocellulolytic enzyme production in wastewater treatment systems
Source: Biotechnol Rep (Amst). 2025 Jan 10;45:e00875. doi: 10.1016/j.btre.2025.e00875 (PMC11791311; doi:10.1016/j.btre.2025.e00875)

Table S1. Parameters of secondary wastewater used for the harvesting tests

Parameter	Value		
BOD_5 (biochemical oxygen demand)	5,8	± 0.62	mg/L
COD (chemical oxygen demand)	38	± 3.61	mg/L
SS (Suspended solids)	6	± 1.26	mg/L
NH_4-N (dissolved ammonium)	2,04	± 0.11	mg/L
NO_2-N (dissolved nitrite)	0,05	± 0.01	mg/L
NO_3-N (dissolved nitrate)	3,11	± 0.14	mg/L
PO_4-P (dissolved phosphate)	0,29	± 0.02	mg/L
pH	7,5	± 0.10	

Figure S1. Microscopy images of *Tetradesmus obliquus* microalgal cells (a, b) before fungal-assisted harvesting; (c, d) after fungal-assisted harvesting; (e, f) immediately after addition of *I. lacteus* pellets (0 hours of harvesting); (g, h) accumulated by fungal hyphae after 24 hours of fungal-assisted harvesting.

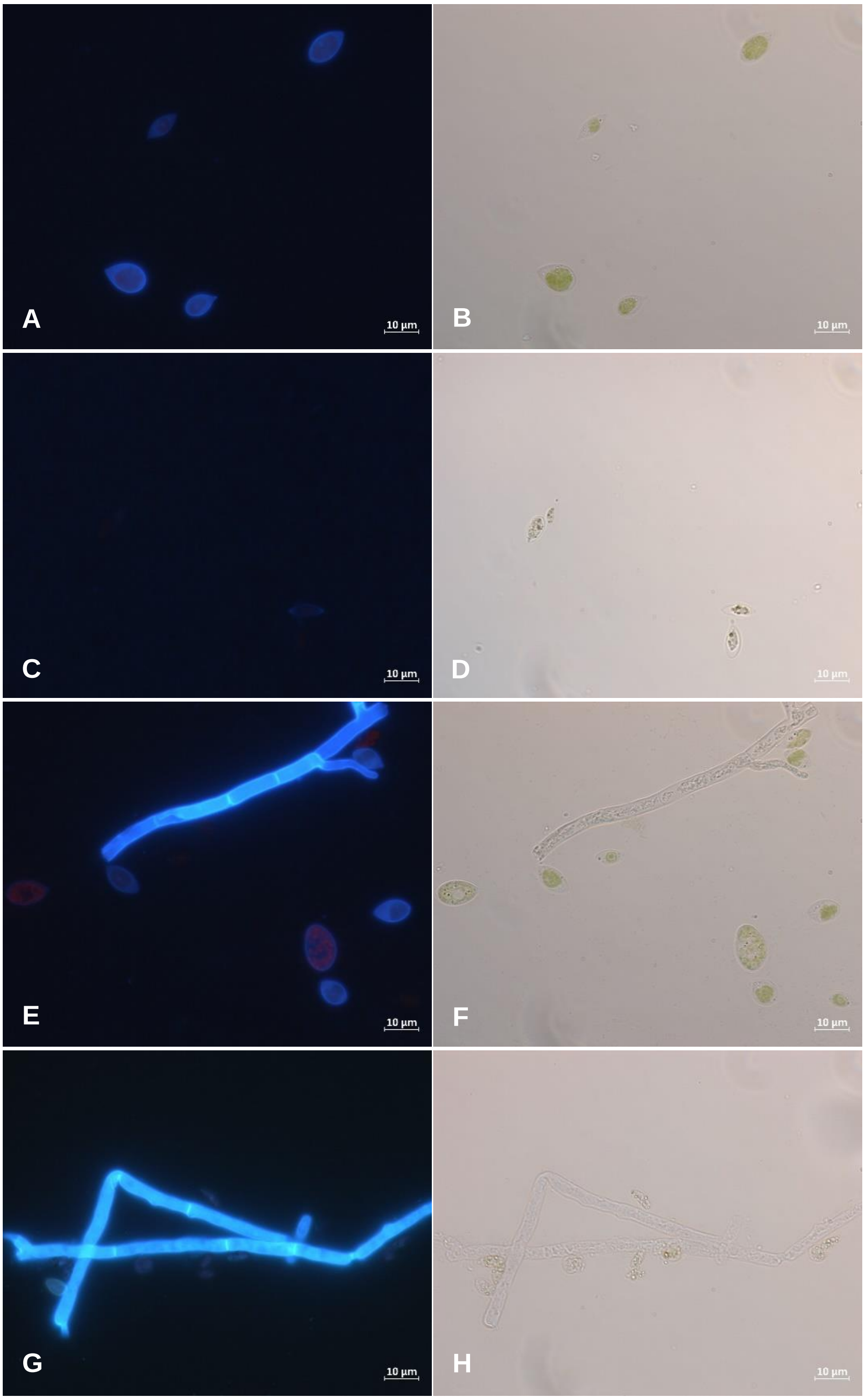

Figure S2. Microscopy images of *Chlorella vulgaris* microalgal cells (a, b) before fungal-assisted harvesting; (c, d) after fungal-assisted harvesting; (e, f) immediately after addition of *I. lacteus* pellets (0 hours of harvesting); (g, h) accumulated by fungal hyphae after 24 hours of fungal-assisted harvesting.

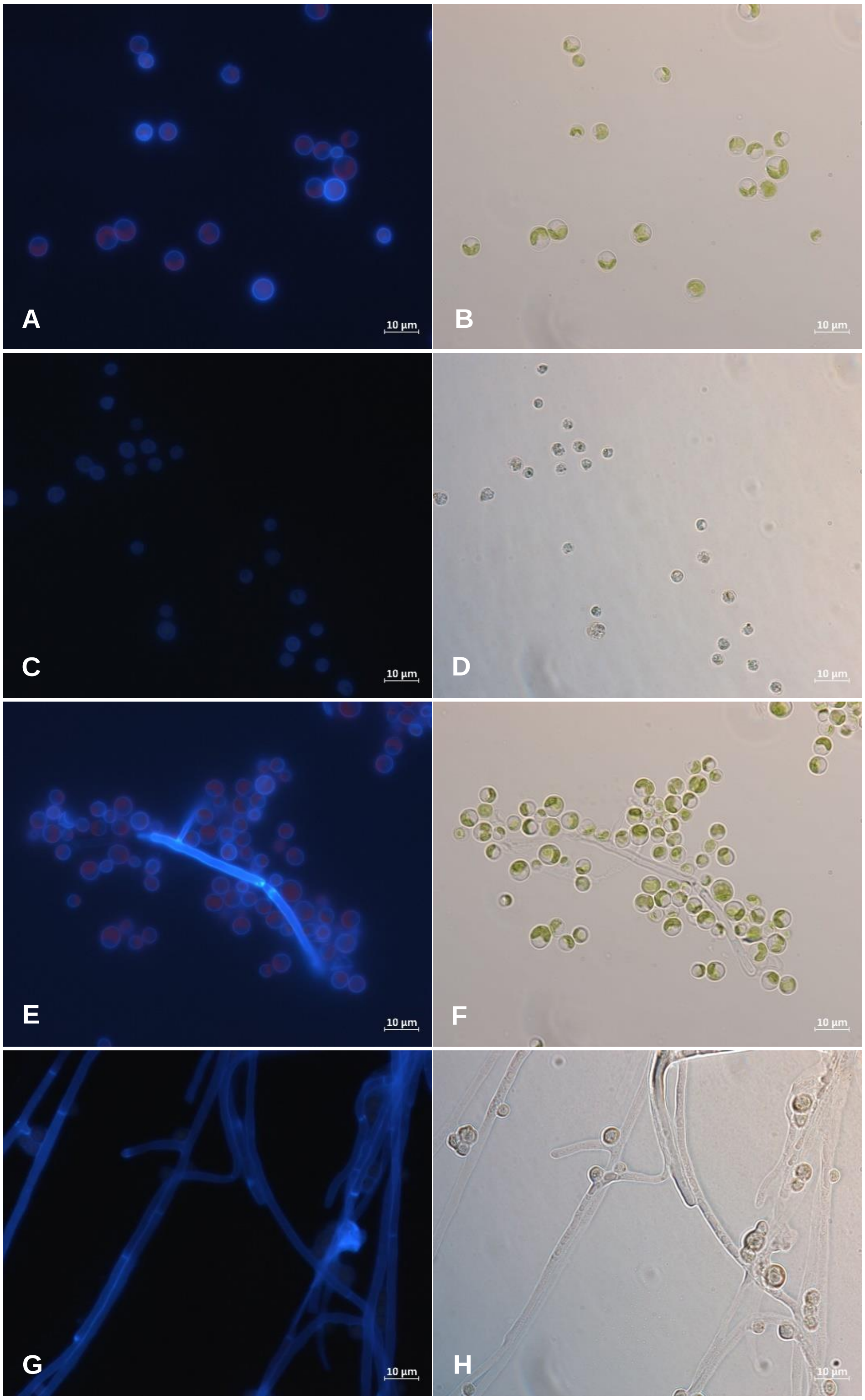

Supplement: Supplementary file 1 [file mmc1.pdf]
